# Clinical outcomes of a novel single-stage cartilage repair technique using calcified cartilage zone debridement with hyalofast

**DOI:** 10.1371/journal.pone.0328978

**Published:** 2025-07-24

**Authors:** Badrul Akmal Hisham Md Yusoff, Muhammad Ikmal Hazli, Norlelawati Mohamad, Muhamad Karbela Reza Ramlan, Nik Kamarul Arif Bin Nik Kamrulzaman, Mohamed Razzan Rameez, Mohamad Azwan Aziz

**Affiliations:** 1 Department of Orthopaedic, Faculty of Medicine, Universiti Kebangsaan Malaysia, Kuala Lumpur, Malaysia; 2 Department of Orthopaedic, Hospital Chancellor Tuanku Mukhriz, Kuala Lumpur, Malaysia; University of Vermont College of Medicine, UNITED STATES OF AMERICA

## Abstract

**Introduction:**

This study aims to determine the clinical outcomes of a new technique of cartilage repair surgery, using calcified cartilage zone debridement coupled with Hyalofast and bone marrow aspirate concentrate, in terms of pain and functional outcomes.

**Methods:**

This was a retrospective case series in 19 patients with cartilage injuries, ICRS 3 and 4. Using arthroscopic examination, cartilage defects were identified and debrided. Then, the calcified cartilage zone was identified and scrapped, until punctate bleeding occurred. Hyalofast was fixed into the defect and bone marrow aspirate concentrate was injected into the Hyalofast. Patients were followed up at baseline, 1, 6, and 18 months post-operative, using visual analog scale, KOOS, IKDC, and Lysholm Score.

**Results:**

The mean age was 45.33 ± 9.68 years, with the mean cartilage defects of 10.21 ± 11.10 X 9.43 ± 10.85 mm^2^. Among the cohort, seven patients (36.8%) underwent three chondral repair procedures, five (26.4%) received two procedures, and the remaining seven (36.8%) were treated with a single procedure. In the KOOS subscale, there was a steady improvement; symptoms (mean difference: −23.87, CI: −43.97 – −3.77, p-value = 0.015), pain (mean difference: −28.39, CI: −43.94 – −12.83, p-value = 0.001), activity of daily living (mean difference: −26.23, CI: −40.95 – −10.14, p-value = 0.001), and sports subscale (mean difference: −57.36, CI: −80.76 – −33.97, p-value < 0.001).

**Conclusion:**

The calcified cartilage zone debridement technique served as a novel technique to preserve subchondral plate allowing better outcomes for cartilage repair.

## Introduction

Articular cartilage, the specialized tissue that provides a smooth, low-friction surface within our joints, presents a challenge of healing when injured. Its avascular nature, coupled with the limited capacity of chondrocytes to proliferate and repair, hinders natural healing processes [1]. Consequently, cartilage injuries often progress to osteoarthritis (OA), a debilitating condition characterized by pain, stiffness, and loss of joint function [1].

While various cartilage repair techniques exist, achieving consistent and durable regeneration of hyaline cartilage, the biomechanically superior type remains a significant hurdle. This difficulty underscores the ongoing pursuit of innovative therapies, one of which is Hyalofast, a promising scaffold-based approach [2]. The common techniques used in cartilage repair surgery are using scaffold engineering with bone marrow stimulation techniques such as activated bone marrow aspirate, microfracture, and drilling, aiming to stimulate chondrocyte cell differentiation [3,4]. Looking at a different perspective, subchondral plate preservation has regained its spotlight again in cartilage repair surgery for its role in the pathogenic process, as well as necessity to consider this structure in the treatment of chondral injury. While microfracture remains a frequently employed bone marrow stimulation technique within scaffold-based tissue engineering, our clinical observations consistently reveal a notable correlation between the procedure and significant postoperative morbidity, characterized by pronounced pain and accelerated progression to osteoarthritis. A systematic review examining 727 microfracture cases, reported that microfracture, when applied to chondral defects of the knee measuring 2–4 cm², exhibits a substantial propensity for osteoarthritis progression [5]. This is coupled with suboptimal chondral defect repair and limited restoration of pre-injury athletic function. Reoperation rates, reported range from 2.9% to 41%, underscore a discernible trend of diminishing clinical efficacy over time [5]. Other studies have shown that patients treated with microfracture led to early subchondral bone changes such as formation of subchondral bone cysts and intralesional osteophytes [6,7]. This raised concerns that such procedure could negatively impact the outcomes of cartilage repair. One theory suggests that marrow stimulation technique activates secondary centres of ossification at the subchondral bone plate, initiating osteoarthritis [8]. Several studies demonstrated a 27–33% incidence of intralesional osteophyte and thickening of subchondral plate [6,7]. In addition, preserving subchondral plate in animal studies has shown that debridement of calcified cartilage zone results in better healing of articular cartilage defects [9,10].

Given the limitations of traditional marrow stimulation particularly the production of inferior fibrocartilage and inconsistent healing, there is a clear need for approaches that preserve the subchondral bone and encourage the formation of stronger, hyaline-like cartilage. Thus, we introduced a cartilage repair technique using calcified cartilage zone debridement coupled with Hyalofast without microfracture for marrow stimulation. Therefore, this study aims to determine the clinical outcome of a cartilage repair technique using calcified cartilage zone debridement coupled with Hyalofast and bone marrow aspirate concentrate, in terms of pain and functional outcomes.

## Materials and methods

### Study design

This study was a retrospective case series, single centre study done at Orthopaedics & Traumatology Department, Hospital Canselor Tuanku Mukhriz, Malaysia, that included all adults aged 18 years old and above. Sample was recruited using a purposive sampling method from the arthroscopic registry. Inclusion criteria included International Cartilage Repair Society (ICRS) Grade 3 or 4 cartilage lesions based on MRI, confirmed with diagnostic arthroscopic of the affected knee, from 1^st^ January 2022. Exclusion criteria included were incomplete data set. Demographic data including age, sex, pre-morbid, and smoking status, were recorded in arthroscopic registry. Intraoperative findings were recorded including location of injury, grading of cartilage injury, concomitant injuries, and concomitant procedures in arthroscopic registry. Post-operative clinical outcomes using Knee Injury and Osteoarthritis Outcome Score [KOOS], International Knee Documentation Committee score (IKDC), and Lysholm knee scoring were recorded in arthroscopic registry. All records stored in the clinical arthroscopic registry 1^st^ January 2022–31^st^ December 2023 were included in this study. Data collection started from 1^st^ January 2024–1^st^ May 2024. This study was conducted in accordance with the Declaration of Helsinki and Good Clinical Practice. Ethics approval was applied and obtained from the Ethics and Research Committee [JEP-2023–672]. Written and informed consent were obtained from the patients to use the information before the commencement of the study during their follow-up.

## Technique description

### Diagnostic arthroscopy

The surgeries were performed by two credentialed sports surgeons at the centre who have more than 5 years of experience in arthroscopic knee surgery. Standard anterolateral (AL) and anteromedial (AM) portals were established adjacent to the patellar tendon. Diagnostic arthroscopy was performed via the AL portal with a 30° scope. Cartilage lesions were evaluated and graded per the ICRS classification. Concurrent soft tissue injuries, including meniscal and ligamentous pathology, were documented in arthroscopic registry. The size of cartilage defect was measured using the tip of the probe.

### Cartilage defect preparation

**Fig 1:**
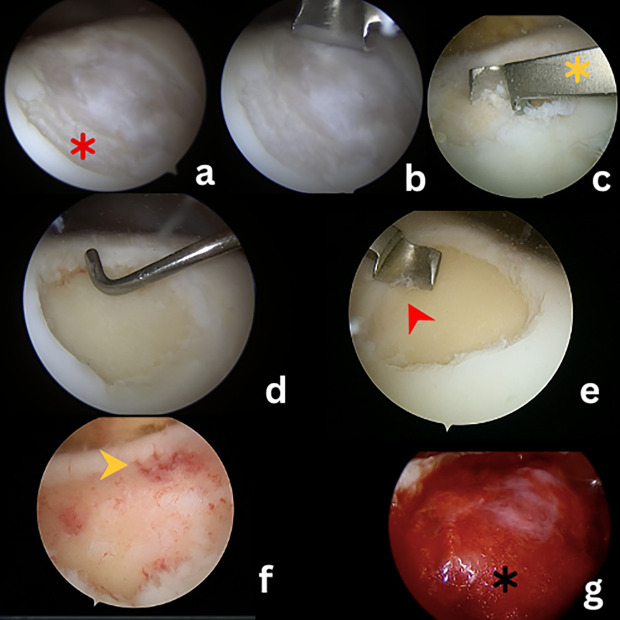
Steps for calcified cartilage zone debridement with Hyalofast. (a) identify the cartilage injury (red asterisk), (b and (c) cartilage is debrided until subchondral bone is exposed using chondrectome (yellow asterisk), (d) cartilage bed is measured **(e)** CCZ layer is identified as white shiny layer (red arrowhead), and is removed using condrectome until punctate bleeding (yellow arrow head) is seen as image **(f)**, **(g)** Hyalofast (black asterisk) is placed onto the cartilage bed, and fibrin sealant is used to fix the scaffold after bone marrow aspirate concentrate injected into the scaffold.

Using a chondrectome, the cartilage defect site was debrided and scrapped until a healthy cartilage border could be achieved. The cartilage was debrided until a calcified cartilage zone identified. Calcified cartilage zone was seen white thin layer above the subchondral bone. This layer was scrapped off using chondrectome until punctate bleeding was seen. Then, the bed was remeasured for the scaffold placement using tip of the probe. This technique is shown in Fig 1.

### Scaffold preparation

The Hyalofast scaffold was cut to the dimensions of the defect measured. Prior to the insertion of scaffold, intra-articular fluids were removed for scaffold fixation. Scaffold was introduced intra-articularly via half-pipe cannula and positioned within the lesion using a probe.

### Bone marrow harvest

Bone marrow was harvested concurrently. Anterior superior iliac spine was identified using palpation, an incision was made at the iliac crest around 1 cm. Marrow Cellution^TM^ bone marrow aspiration system, 11-gauge or 13-gauge diameter was used for bone marrow aspiration, without needing centrifugation [11]. A two mls of heparin was withdrawn and mixed with eight mls of saline and was used to flush the ten mL syringe. A 1 ml of mixture of heparin-saline was left inside the ten mL syringe, equivalent to 2000 units of heparin. After disinfectant, Marrow Cellution^TM^ was driven through the periosteum until reached bone marrow. Using the syringe flushed with heparin-saline, ten mls of bone marrow aspirated. Then, bone marrow aspirate concentrate was injected into the scaffold using 18-gauge branula. The scaffold then stabilized using Tisseel fibrin sealant, for scaffold fixation.

### Postoperative protocol

Post-rehabilitation plays a vital role in the effective management of cartilage repair. Following surgical intervention, the standard protocol for cartilage repair in weight-bearing regions typically involves strict non-weight-bearing for six weeks. However, our institution has adapted the ambulation technique, implementing heel-touch ambulation rather than the conventional toe-touch approach. This modification promotes the reactivation of the proprioceptive nervous system, enhances eccentric quadriceps contraction, facilitates early knee extension, and reduces the risk of arthrofibrosis.

For lesions involving the patellofemoral joint (patella facet and trochlea), a more conservative rehabilitation strategy is employed, particularly concerning range of motion (ROM) and patellar mobility. Rehabilitation begins with a graded ROM protocol, initially restricting movement to 0–30 degrees during the first two postoperative weeks, followed by gradual progression. This approach optimizes healing, minimizes postoperative pain and swelling, and improves outcomes, particularly within the critical first six weeks.

Nevertheless, individualized adjustments are made based on intraoperative findings, patient-specific factors, and functional demands, ensuring a tailored rehabilitation plan for optimal recovery. Detail rehabilitation program is outline in Table 1.

**Table 1:** Post-operative rehabilitation strategies. This is a general guide on the management of the rehabilitation. However, the prescription is often individualize based on patient’s condition, intra-operative findings and patient’s demand.

	REHABILITATION	DURATION OF REHAB	MANAGEMENT	SPECIAL PRECAUTION
PHASE I	Reduce swelling	0-6 week	isometric quads contraction cryo cuff ankle pump	
	Muscle activation	0-6 week	isometric quads contraction	
	Muscle strengthening	0-6 week	straight leg raise exercise in 4 directions -free weight short arc knee extension – free weight	
	range of motion	0-2 week	0-30degree	*gentle precaution for patella mobility in repaired patella defect
		2-4 week	0-60 degree	
		4-6 week	0-120 degree	
	Ambulation	0-6 week	Heel touch walking for 2 week Then, partial to full weight bearing with cruthes	
PHASE II	Reduce swelling	6-12 week	isometric quads contraction cryo cuff ankle pump	
	Muscle activation	6-12 week	isometric quads contraction	
	Muscle strengthening	6-12 week	straight leg raise exercise in 4 direction -with weight double leg squat – free weight to light weight lunges – free weight to light weight single leg extension – free weight to light weight	
	range of motion	6-12 week	aim for full range of motion cycling	
	Ambulation	6-12 week	partial to full weight bearing ambulation	
	Proprioception	6-12 week	single leg balance exercise	
PHASE II	Muscle strengthening	>12 week	double leg squat – progressively increasing weight lunges – progressively increasing weight single leg extension – progressively increasing weight leg press – progressively increasing weight resistance cycling	
	range of motion	>12 week	maintain full range of motion	
	Ambulation	>12 week	gait retraining aiming for normal gait	
	Proprioception	>12 week	dynamic balance exercise single leg balance on uneven surfaces	

### Follow up

Pain was assessed using Visual Analog Score (VAS). Functional outcomes were assessed using the Lysholm knee scoring scale, KOOS, and the IKDC scores.

### Statistical analysis

Functional outcome scores were compared to assess statistical significance, with p-values of less than 0.05 considered significant. Shapiro Wilks test was used to test for normalities within the groups and reported a normal distribution. Descriptive analysis was used. Age and weight were reported as mean and standard deviation while other demographic data were reported as frequency and percentage. Mauchly’s Test of Sphericity was performed using general linear model on KOOS subset, IKDC, VAS score, and Lysholm score at baseline, 1-month, 6-month and 18-month follow-up. Mauchly’s test was used to determine the sphericity. If Mauchly’s test value > 0.05, it indicates normal sphericity, and repeated ANOVA test was performed. If Mauchly’s test value < 0.05, it violates the sphericity, and correction were done using Greenhouse-Geisser. The normality testing between single cartilage procedure group with multiple cartilage procedures group using Shapiro wilks were performed, resulting p-value < 0.05. The outcome of KOOS subset and VAS score were compared between groups using Mann-Whitney test. All statistical analyses were conducted using SPSS software version 26.

## Results

The present study’s sociodemographic profile of the participants included 19 participants, of which nine were female (47.37%) and ten were male (52.63%). The mean age was 45.33 ± 9.68 years. The mean weight was 77.11 ± 17.08 kg. Only one patient (5.2%) was an active smoker. Seven patients (36.8%) had X-ray findings of knee osteoarthritis, Kellgren and Lawrence grade I, and three patients (15.7%) had knee osteoarthritis, Kellgren and Lawrence grade II. Intraoperatively, the mean cartilage defect were 10.21 ± 11.10 X 9.43 ± 10.85 mm^2^. Three patients (15.7%) had partial anterior cruciate ligament tear, and one patient (5.2%) had medial meniscus tear. Among the cohort, seven patients (36.8%) underwent three chondral repair procedures, five (26.4%) received two procedures, and the remaining seven (36.8%) were treated with a single procedure One patient (5.2%) had concomitant partial meniscectomy procedure. Demographic data are outlined in Table 2.

**Table 2:** Baseline demographic and intra-operative findings.

Baseline data	Descriptive analysis
**Gender**	
Male	10 (52.63)*
Female	9 (47.37)*
**Age (years)**	45.33 (9.68)^
**Weight (kg)**	77.11 (17.08)^
**Race**	
Malay	19 (100)*
Comorbid	
Diabetes	2(10.5)*
No Comorbid	17 (89.5)*
Smoking status	
Active smoker	1 (5.2)*
Non-smoker	18 (94.8)*
Knee X-ray	
Normal X-ray	10 (47.5)*
KL 1	7 (36.8)*
KL 2	3 (15.7)*
KL 3	0 (0)*
KL 4	0 (0)*
Location of chondral injury	
Right Subchondral Injury	13 (68.4)*
Left Subchondral Injury	6 (31.6)*
**Site of Operation**	
Right	13 (68.4)*
Left	6 (31.6)*
Location of chondral injury	
Medial femoral condyle	15 (39.4)*
Lateral femoral condyle	3 (7.9)*
Patella	9 (23.7)*
Trochlear	11 (30)*
Concomitant injury	
Anterior cruciate ligament tear	3 (15.7)*
Medial Meniscus tear	1 (5.2*

Table 2 demonstrates the baseline characteristic pre-operative demographic and intra-operative findings.

* frequency (percentage).

^ mean (standard deviation).

KL = Kellgren Lawrence grading (Grading for knee osteoarthritis).

**Fig 2:**
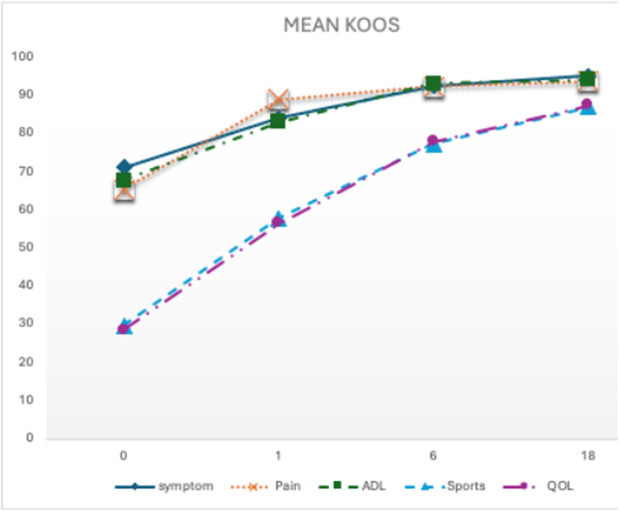
Clinical improvement of single-stage cartilage repair using calcified cartilage zone debridement with Hyalofast using KOOS subscale; symptom, pain, activity of daily living (ADL), Sports, and Quality of life (QOL). Pre-operatively, for KOOS- Symptoms, the mean score was 71.24 ± 27.3, and 95.11 ± 11.67 at 18 months post-operation (p-value = 0.004). For the KOOS pain subscale, the mean score was 65.37 ± 17.93 pre-operatively, and 93.76 ± 15.02 at 18 months post-operation (p-value < 0.001). For the KOOS function of activity of daily living subscale, the mean score was 67.87 ± 19.71 pre-operatively, and 94.11 ± 14.06 at 18 months post-operation (p-value < 0.001). For the KOOS sports subscale, the mean score was 29.73 ± 22.01 pre-operatively, and 87.11 ± 25.6 at 18 months post-operation (p-value < 0.001). For the KOOS quality of life subscale, the mean score was 28.61 ± 22.75 pre-operatively, and 87.50 ± 26.84 at 18 months post-operation (p-value < 0.001).

In the KOOS Symptoms subscales, there was a steady improvement over time. Pre-operatively, the mean score for KOOS-Symptom was 71.24 ± 27.3, and 95.11 ± 11.67 at 18 months post-operation (mean difference: −23.87, CI: −43.97 – −3.77, p-value = 0.004). For the KOOS pain subscale, the mean score was 65.37 ± 17.93 pre-operatively, and 93.76 ± 15.02 at 18 months post-operation (mean difference: −28.39, CI: −43.94 – −12.83, p-value < 0.001). For the KOOS function of activity of daily living subscale, the mean score was 67.87 ± 19.71 pre-operatively, and 94.11 ± 14.06 at 18 months post-operation (mean difference: −26.23, CI: −40.95 – −10.14, p-value < 0.001). For the KOOS sports subscale, the mean score was 29.73 ± 22.01 pre-operatively, and 87.11 ± 25.6 at 18 months post-operation (mean difference: −57.36, CI: −80.76 – −33.97, p-value < 0.001). For the KOOS quality of life subscale, the mean score was 28.61 ± 22.75 pre-operatively, and 87.50 ± 26.84 at 18 months post-operation (mean difference: −58.88, CI: −80.46 – −37.28, p-value < 0.001). KOOS score improvement is shown in Fig 2 and Table 3.

**Table 3:** Pre-operation and post-operation clinical outcomes.

	Baseline	1-month Post-operative	6-month Post-operative	18 -onth Post-operative	Mean difference	CI	Mauchly’s W	P value	F	df	η²
KOOS- symptom	71.24 ± 27.3	84.02 ± 10.99	92.66 ± 11.63	95.11 ± 11.67	−23.87	−43.97 – −3.77	0.021	0.04^	9.13	1.29,23.2	0.337
KOOS – pain	65.37 ± 17.93	89.05 ± 11.71	92.38 ± 13.97	93.76 ± 15.02	−28.39	−43.94 – −12.83	0.067	<0.001*	20.22	3,54	0.529
KOOS- ADL	67.87 ± 19.71	83.04 ± 10.08	93.11 ± 13.2	94.11 ± 14.06	−26.23	−40.95 – −10.14	0.008	<0.001^	22.01	1,19.7	0.550
KOOS- Sports	29.73 ± 22.01	57.89 ± 16.94	77.36 ± 24.2	87.11 ± 25.6	−57.36	−80.76 – −33.97	0.072	<0.001*	44.72	3,54	0.713
KOOS -QOL	28.61 ± 22.75	56.57 ± 19.14	77.96 ± 25.03	87.50 ± 26.84	−58.88	−80.46 – −37.28	0.060	<0.001*	58.78	3,54	0.766
VAS score	7.52 ± 2.01	4.63 ± 2.09	2.58 ± 2.30	1.57 ± 2.755	5.94	3.5–8.3	0.198	<0.001*	39.88	3,54	0.689
Lysholm	53.82 ± 3.73	71.36 ± 2.29	90.26 ± 3.35	95.58 ± 3.27	−38.73	−54.5 – −22.7	0.048	<0.001^	52.17	1.2,21.9	0.743
IKDC	33.21 ± 15.95	50.63 ± 12.1	69.68 ± 18.2	72.36 ± 18.8	−39.15	−54.5–23.83	0.028	<0.001^	57.88	1.14,20.6	0.763

Table 3 showed a repeated general linear model test comparing clinical outcomes at baseline, 1-month post -perative, 6-month post-operative and 18-month post-operative. Mauchly’s test was used to determine the sphericity. If Mauchly’s test value > 0.05, it indicates normal sphericity and repeated ANOVA tests were performed, indicated by *. If Mauchly’s test value < 0.05, it violates the sphericity, and correction were done using Greenhouse-Geisser indicated by ^. P value < 0.05 indicates a significant test.

ADL: Activity of daily living.

IKDC: International Knee Documentation Committee.

KOOS: Knee Injury and Osteoarthritis Outcome Score.

QOL: quality of life.

The VAS score, showed a substantial reduction over time as in Table 3. The Lysholm score and IKDC score also demonstrated remarkable improvements.

When compared between single cartilage procedure (n = 7) lesion with multiple cartilage procedures (n = 12) at baseline and 18-month post-operative, both groups demonstrated a marked improvement in outcomes of KOOS score subset and VAS score, with no statistical differences between two groups as in Table 4

**Table 4:** Comparison of clinical outcomes between single cartilage lesion and multiple cartilage lesions.

Clinical Outcomes	Follow-up	Single cartilage procedure (median ± IQR)	Multiple cartilage procedures (median ± IQR)	P-value
KOOS -symptom	Baseline	75 ± 42.8	80.3 ± 50.8	0.670
	18-month	100 ± 7.14	100 ± 2.6	0.355
KOOS- Pain	Baseline	76.3 ± 21.1	61.8 ± 23.3	0.470
	18-month	100 ± 13.6	100 ± 1.9	0.305
KOOS- ADL	Baseline	76.47 ± 11.7	66.9 ± 20.9	0.090
	18-month	100 ± 7.3	100 ± 1.1	0.413
KOOS- Sports	Baseline	30 ± 35.0	17.5 ± 51.	0.349
	18-month	90 ± 20.0	100 ± 3.7	0.187
KOOS- QOL	Baseline	31.2 ± 43.7	15.6 ± 35.9	0.228
	18-month	81.25 ± 18.7	100 ± 1.2	0.065
VAS Score	Baseline	8 ± 3.0	8 ± 2.7	0.796
	18-month	1 ± 3.0	0.5 ± 1	0.470

Table 4 shows the comparison of KOOS subset and VAS score at baseline and 18-month between single cartilage procedures and multiple cartilage procedures using Mann- Whitney test, and op value < 0.05 is considered significant.

There was zero dropout during subsequent follow-up, and there was no adverse event reported throughout follow-up. Follow-up Xray showed no subchondral bone lesion such as subchondral cyst formation.

## Discussion

This study is the first to assess the clinical outcomes of a single-stage cartilage repair using calcified cartilage zone layer debridement with Hyalofast without needing microfracture. The patient-reported symptom, function, and quality-of-life scores from the self-administered KOOS, IKDC, and visual analog scale pain scales improved clinically and statistically significantly over the 18-months after the surgery. The significant improvements in the KOOS scores across all subscales indicate that the patients experienced meaningful enhancements in their knee function and overall quality of life. The marked decrease in pain scores from 1-month to 6-months post-operatively suggests a durable repair process, as patients reported substantial and sustained relief from pain.

Remarkable outcomes from our studies demonstrate a clear superiority over other contemporary cartilage repair techniques. A study by Ryu et al. (2020), using microfacture, Hyalofast, and BMAC to treat cartilage defects, demonstrated improved outcomes in VAS score and IKDC. At 2 years follow-up, the VAS score (0.92 ± 0.98) and IKDC score (80.27 ± 9.48) with mean cartilage defect (4.33 ± 1.66 cm^2^), were comparable to CCZ debridement at 18 month follow-up [12]. A similar study by Gobbi et al. (2016) showed comparable results of IKDC (83 ± 15, and Lysholm score (90 ±  25) at 2 years follow-up, with median lesion size of 6.5 cm^2^

The improvement in KOOS score, IKDC, VAS, and Lysholm score in our study is better compared to matrix-induced autologous mesenchymal stem cell implantation [KOOS- P 93.76 vs 88.1 at 24 months] [13], matrix-induced autologous chondrocyte implantation [KOOS-P 93.76 vs 82.54 at 24 months] [13], autologous matrix-induced chondrogenesis (AMIC) using a cell-free hyaluron-based scaffold and microfracture technique [IKDC 69.68 VS 54.58 at 6 month] and [IKDC 72.36 VS 66.19 at 12 months] [14], AMIC with leucocyte–platelet-concentrated membrane [VAS 2.58 VS 3.3 to 3.6 at 6 months] [15], and human culture-expanded autologous bone marrow mesenchymal stem cells transplanted on platelet-rich fibrin glue [Lysholm score 95.58 VS 86.0 at 12 months] [16]. Overall, our IKDC scores are comparable to a meta-analysis for scaffold implanted mesenchymal stem cells vs acellular scaffolds with concentrated bone marrow aspirate [mean IKDC 31.7–68.2 and 64.3–92.13], respectively [17]. These results suggest that single-stage cartilage repair using calcified zone layer debridement with Hyalofast without microfracture, may be as effective as, or even more effective than, a single-stage option for patients with articular cartilage defects of the knee compared to other marrow stimulation techniques. The sustained improvements in these scores over 18 months suggest that the calcified cartilage zone debridement combined with Hyalofast may offer a viable alternative to traditional cartilage repair techniques, particularly for patients seeking long-term relief and functional recovery.

Several mechanisms may explain the favorable outcomes observed in this study. The preservation of subchondral bone integrity is critical, as it provides a stable foundation for the repaired cartilage. By avoiding microfractures or drilling, the calcified cartilage zone debridement technique minimizes disruption to the underlying bone, potentially enhancing the healing environment. Recent interest in the calcified zone of cartilage function has led to the development of our technique. Calcified layer of articular cartilage consists of dispersed, hypertrophic chondrocytes within lacunae in calcific matrix, which function as force transmission, assist in biochemical communication, and act as physical barrier to subchondral bone [18]. Interestingly, one of the discovered repaired mechanisms in early osteoarthritis is the formation of microcracks in the calcified cartilage layer, which leads to vascular invasion and promotes healing, similar to punctate bleeding in our technique [8,19,20]. This unique adaptive mechanism led to recruitment of osteocytes and maintaining cartilage integrity. In an animal study, removal of calcific cartilage layer appears to be associated with both an optimal quantity of reparative tissue and its secure integration [21]. Moreover, preserving subchondral bone plate is advantageous as i) the subchondral bone plate acts as a foundation for the newly formed cartilage. It provides mechanical support and stability, allowing the repair tissue to withstand physiological loads and preventing it from collapsing or delaminating, ii) subchondral bone marrow contains mesenchymal stem cells and growth factors that are essential for cartilage regeneration as it allows these cells and factors to migrate to the defect site and promote healing, iii) subchondral bone plate provides a scaffold for the newly formed cartilage to integrate with the surrounding native tissue which is crucial for the long-term success of the repair, as it ensures proper load transfer and prevents the formation of a weak interface that could lead to failure, and iv) damage to subchondral bone plate can lead to complications such as subchondral cysts, bone necrosis, and delayed healing [22,23]. One of the primary disadvantages of marrow stimulation techniques using microfracture is the quality of the repair tissue that is generated. Studies have consistently shown that the repair tissue resulting from these procedures is predominantly fibrocartilage rather than hyaline cartilage, which is the desired outcome for optimal joint function [5,24].

### Limitations

Most of the cohort patients had small to medium size defects, with mean size of 10.21 ± 11.10 x 9.43 ± 10.85 mm^2.^, with advanced stage of articular disease, showed promising results with this technique. Despite the promising results, this study is not without limitations. This study has several key limitations. First, the small sample size (n = 19) and single-center design may restrict the generalizability of our findings to broader populations. Second, the absence of imaging outcomes limits our ability to objectively evaluate structural cartilage repair, despite evidence suggesting weak correlations between imaging and clinical healing. Though post-operative magnetic resonance imaging (MRI) is theoretically ideal for cartilage healing assessment, meta-analyses reveal a lack of correlation between MRI findings and actual healing [25]. Furthermore, experts in cartilage musculoskeletal radiologist is required to interpret specific outcome measures which is MOCART (magnetic resonance observation of cartilage repair tissue) scores to assess post-operative cartilage regeneration [26]. Thus, it is not used as an outcome measure in our study. Third, the 18-month follow-up period may be insufficient to assess long-term complications, such as osteoarthritis progression or late repair failure. Finally, the uncontrolled pre-post design cannot exclude regression to the mean or natural fluctuations as contributors to the observed improvements. Future multicenter randomized trials with larger cohorts, longer follow-ups, and standardized imaging protocols are needed to validate these preliminary results.

## Conclusion

Overall, the present study shows that calcified cartilage zone debridement combined with Hyalofast and bone marrow aspirate concentrate substantially relieves pain and improves function in patients with knee cartilage injuries. The sustained improvements in VAS, Lysholm, and IKDC scores underscore the promise of this technique as a durable alternative to traditional marrow stimulation methods. As the field of cartilage repair continues to evolve, further research should focus on refining these approaches and evaluating their broader applicability across diverse patient groups. Looking forward, future research should explore the long-term sustainability and expanded applicability of this method, particularly in diverse patient populations, to create a better surgical technique for cartilage repair surgery.
